# Inappropriate ICD Shocks for Inappropriate Reasons

**Published:** 2008-02-01

**Authors:** Rakesh K Pai, Moeen Abedin, David A Rawling

**Affiliations:** Department of Internal Medicine, Cardiology Section, University of Utah Health Sciences Center, Salt Lake City, UT USA

**Keywords:** Inappropriate ICD Shock, DF-1 Lead Terminal Reversal, Integrated Bipolar ICD lead, Complication, External Alternating Current

## Abstract

ICD shocks can result from a variety of etiologies; determining the proper etiology of the inappropriate shock is essential for correction of the problem. Electromagnetic interference (EMI) can mimic cardiac signals and cause inappropriate defibrillator shocks. We present two cases of inappropriate ICD shocks due to EMI and reversal of the proximal and distal DF-1 lead terminals of the ICD lead. These are two unusual etiologies for inappropriate defibrillator shocks.

## Case Discussion

### Case 1

**Figure 1:**
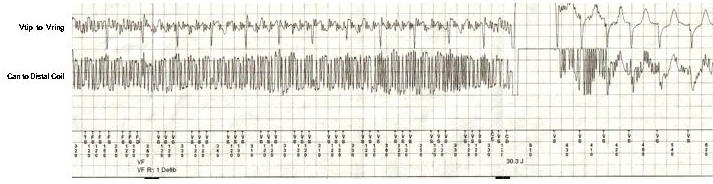
The near field ICD electrogram (V tip to V ring) is present on the top, and the ICD shock electrogram (Can to Distal coil) is on the bottom. Note the high frequency noise present on both sets of electrograms. The patient receives a single 30 Joule shock; however, the noise persists and only begins to dissipate after she receives the shock (last few seconds of the tracing) and begins to swim away from source of alternating current leak.

A 38 year old woman with normal left ventricular function, normal cardiac MRI, and a history of sustained monomorphic ventricular tachycardia at 240 beats per minute (left bundle morphology, superior axis) was implanted with a single chamber Medtronic Maximo VR 7232 (Medtronic Inc, Minneapolis, MN USA) implantable cardioverter-defibrillator (ICD) in 2001. The patient had never received an ICD shock over six years of follow-up. In the summer of 2007, the patient received a single ICD shock. The ICD interrogation (Figure 1) revealed a high frequency 50 Hz artifact on the ventricular channel sensed by the device as ventricular fibrillation, a 30 Joule shock was delivered. There was no evidence of ICD malfunction, with a ventricular sensitivity of 0.3 mV, R waves of 6.1 mV, with ventricular lead and shock impedances of 400 Ohms and 55 Ohms, respectively. Upon further questioning, it was discovered that the patient was swimming within two feet of an underwater pool light.  The light had "shocked" several swimmers in the past.  An electrician confirmed light malfunction due to improper grounding with evidence of current leak into the pool. We report an unusual case of an inappropriate ICD shock caused by the ICD sensing alternating current from an unexpected external source.

### Case 2

**Figure 2:**
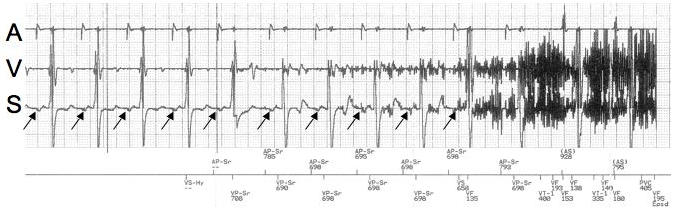
The atrial sense channel electrogram (A) is present on the top, the near field ventricular sense channel (V) is present in the middle tracing, and the ICD shock (S) electrogram is on the bottom. Very high frequency pectoral myopotentials are present and sensed by the device as VF (marker channel) on both the V and S electrograms.  In this particular tracing the episode was nonsustained and did not trigger ICD shock therapy. The presence of p waves and pectoral myopotentials on the shock electrogram is suggestive of reversal of DF-1 lead terminals, allowing for unipolar sensing from distal coil to the ICD can.

A 70 year old man with a history of ischemic cardiomyopathy, nonsustained ventricular tachycardia and sinus node dysfunction was upgraded from a dual chamber pacemaker to a dual chamber Guidant Vitality 2 DR ICD model T165 (Guidant Inc, St Paul, MN USA) in February of 2007. During ICD testing a single 20-Joule reverse polarity shock terminated VF. In June 2007, the patient presented for routine follow-up and during this visit he reported receiving a single ICD shock in March. He felt well and did not seek medical evaluation at that time. The ICD interrogation revealed a high frequency artifact on the ventricular channel sensed by the device as VF, and a single 20-Joule shock was delivered.  In addition there were numerous stored EGMs demonstrating high frequency noise (Figure 2) recognized as VF but none were sustained long enough to trigger ICD therapy.  There was no evidence of malfunction; R waves were 24 mV, capture threshold of 0.6 V at 0.5 ms, and the ventricular lead and shock impedances were 706 Ohms and 50 Ohms, respectively. The noise could not be reproduced by isometric maneuvers or with pocket manipulation.

## Discussion

Inappropriate ICD shocks can be caused by SVT, lead fracture, electromagnetic interference, and set screw malposition. EMI can arise from the normal functioning of electrical appliances or from alternating current leak [1,2]. The Guidant ICD lead is an integrated bipolar lead, with ventricular sensing from the distal tip to distal coil. In the second case, the proximal and distal ICD DF-1 pins were reversed in the header, resulting in a sensing circuit from the distal coil to the can.  This broad unipolar sensing configuration allowed the detection of pectoral myopotentials, precipitating an inappropriate ICD shock [3,4]. A diagnostic clue for this etiology of inappropriate shock is the presence of p waves (Figure 2; arrows) on the ICD shock EGMs. The high frequency noise artifact is unlikely to be due to EMI, as there is no noise seen on the atrial lead. Furthermore, lead fracture is less likely given adequate sensing and capture thresholds with normal lead impedances. Surprisingly, the patient was successfully defibrillated at initial implant; however, the shock energy pathway from the proximal SVC coil to the distal coil and ICD generator can shunt energy away from the ventricles and is unproven in cardioverting ventricular arrhythmias. The patient was taken to the electrophysiology laboratory and proximal and distal DF-1 pin reversal was confirmed. The DF-1 distal and proximal lead terminal positions were corrected and VF was terminated with a single reverse polarity 14-Joule shock. The patient went home the same day and had no further complications. We present two unique cases of inappropriate ICD shocks due to EMI from an unexpected source alternating current and reversal of the proximal and distal DF-1 lead terminals of the ICD lead.
